# LncRNA NR_136400 Suppresses Cell Proliferation and Invasion by Acting as a ceRNA of TUSC5 That Is Modulated by miR-8081 in Osteosarcoma

**DOI:** 10.3389/fphar.2020.00641

**Published:** 2020-05-15

**Authors:** Liyun Liu, Mingxia Zheng, Xinwei Wang, Yanzheng Gao, Qingguo Gu

**Affiliations:** ^1^Henan Provincial People’s Hospital, School of Clinical Medicine, Henan University, Zhengzhou, China; ^2^Luoyang Orthopedic Hospital of Henan Province, Orthopedic Hospital of Henan Province, Zhengzhou, China; ^3^Department of Paediatrics, Tongren Hospital, Shanghai Jiao Tong University School of Medicine, Shanghai, China; ^4^Department of Spine Surgery, Changzheng Hospital, The Second Military Medical University, Shanghai, China

**Keywords:** long noncoding RNA, osteosarcoma, miRNA, TUSC5, invasion

## Abstract

Long non-coding RNAs (lncRNAs) are emerging as important regulators of the processes involved in cancer development and progression. The molecular mechanism by which lncRNAs regulate the progression of osteosarcoma has not been clearly elucidated. The role of NR_136400, which is an uncharacterized lncRNA, has not been previously reported in osteosarcoma (OS). In the present study, we demonstrated that NR_136400 was downregulated in OS cells and that its downregulation promoted OS cell proliferation, apoptosis, and invasion. NR_136400 downregulation facilitated EMT by inhibiting the expression of E-cadherin and elevating the expression of ZEB1, Snail, and fibronectin. *In vivo* experiments using a xenograft tumor mouse model revealed that NR_136400 downregulation promoted tumor growth in OS. Mechanistic investigations demonstrated that NR_136400 competitively bound to miR-8081 and then upregulated the protein expression of TUSC5. Taken together, a newly identified regulatory mechanism of the lncRNA NR_136400/miR-8081/TUSC5 axis was systematically studied in OS, providing a promising target for therapeutic treatment.

## Background

Osteosarcoma (OS) is a highly malignant bone cancer that originates from bone marrow mesenchymal stem cells. It often occurs in children and adolescents (Mirabello et al., 2009). Surgery combined with chemotherapy or radiotherapy is the basic treatment strategy. Although surgical methods and therapeutic medicines have been modified to improve the survival rate and prognosis of OS patients, the rates of death and metastasis remain high (Zheng et al., 2018). Therefore, identifying the mechanisms underlying OS initiation and progression could be very helpful in finding promising therapeutic strategies and improve clinical outcomes.

Long noncoding RNAs (lncRNAs) are defined as non-protein coding RNAs with transcripts greater than 200 nucleotides in length (Hua et al., 2019). Increasing evidence shows that lncRNAs play important regulatory effects in the physiological and pathological processes of cancer (Bhan et al., 2017; Tehrani et al., 2018). LncRNAs play a vital role in regulating the expression of genes by mediating their transcription, translation, and protein functions (Wang and Chang, 2011; Hu et al., 2018; Begolli et al., 2019). There are several mechanisms that lncRNAs implicate in the process of tumorigenesis: alternative splicing and epigenetic, transcriptional, and post-transcriptional modifications (Li et al., 2019; Shang et al., 2019). The elucidation of the mechanisms of lncRNAs may reveal potential therapeutic targets for the diagnosis and treatment of OS.

The competing endogenous RNA (ceRNA) regulatory network is a newly discovered mechanism for regulating the interaction between RNAs *in vivo* and the function of coding genes, which also expands the previous understanding of a large number of noncoding RNAs *in vivo* (Li et al., 2018). Cancer is still an insurmountable problem that hinders human health. A large number of experiments have confirmed that the ceRNA network exists widely among different tumors, and it may have an impact on the occurrence, development, and prognosis of tumors; in addition, it could become a target for early diagnosis, prognosis evaluation, and tumor treatment (Zhang et al., 2018; Li et al., 2020). At present, the regulatory function of ceRNA has been widely recognized, but whether there are other forms of ceRNA or other regulatory mechanisms remains to be determined. Overall, the role of ceRNA in tumors cannot be ignored, and it is worth further study.

MiRNAs are small noncoding RNAs that are involved in post-transcriptional regulation. The expression of miRNAs in tumors and its implications in the regulation of tumorigenesis and in the early detection, treatment, and prognosis of tumors have been hot topics in cancer research (Zhang et al., 2015). In recent years, miRNAs have been found to play an important role in tumor cell migration, invasion, differentiation, proliferation, and apoptosis (Kushlinskii et al., 2016).

Tusc5 (tumor suppressor candidate 5), also known as lost1 or bec-1, was first found in the study of some gene deletions in lung cancer, and its main function was to inhibit the proliferation of tumor cells (Knotts et al., 2009). The current study confirms that tusc5 expression is significantly present in rodent- and human-specific tissues, especially in mature white and brown fat cells, and peripheral afferent nerves (Oort et al., 2007).

This study identified lncRNA NR_136400, which was uncharacterized in the past, and investigated its biological function in OS cells and the mechanism by which it suppresses cell proliferation and invasion; it was found that lncRNA NR_136400 acts as a ceRNA of TUSC5 that is modulated by miR-8081 to regulate OS formation and progression.

## Materials and Method

### Cell Culture

Four OS cell lines, U2OS, Saos-2, MG-63 & HOS, as well as human bone marrow stem cells (hBMSCs), human foreskin fibroblast-1 (HFF-1) cells, and human osteoblasts (hFOB1.19 cells), were obtained from the Cell Bank of Chinese Academy Sciences (Shanghai, China). All cells were cultured at 37°C in a humidified atmosphere containing 5% CO2 in Dulbecco’s modified Eagle’s medium (DMEM) supplemented with 10% foetal bovine serum (FBS), 100 U/ml penicillin, and 100 μg/ml streptomycin (all from Gibco, Carlsbad, CA, USA).

### RNA Isolation and qRT-PCR

RNA from cell lines was isolated with the use of TRIzol^®^ Reagent (Invitrogen, Carlsbad, CA, USA). One microgram of total RNA was used for the synthesis of cDNA according to the protocol of the Reverse Transcription Kit (Takara, Dalian, China). To quantitate lncRNA NR_136400 and TUSC5 expression, qPCR was carried out with a SYBR^®^ Premix Ex TaqTM II Kit (Takara, Dalian, China). The expression levels of lncRNA NR_136400 and TUSC5 were normalized to those of GAPDH. qRT-PCR was performed using the miScript SYBR Green PCR Kit (Qiagen, Hilden, Germany) to determine miR-8081 expression. The expression level of miR-8081 was normalized to that of U6. All the data were analyzed by the 2^−ΔΔCt^ method.

### Cell Transfection

For overexpression, the NR_136400 overexpression vector was established using the pLVX-IRES-puro vector backbone served by Sangon Biotech Co., Ltd. For knockdown, short-hairpin RNA (shRNA) targeting NR_136400 was used to establish NR_136400-silenced cell lines. U2OS and Saos-2 cells were transfected with 100 nM shRNA using Lipofectamine^®^ 2000 reagent according to the manufacturer’s protocol (Invitrogen; Thermo Fisher Scientific, Waltham, MA, USA). The following experiments were performed using cells harvested at 48 h post-transfection.

### CCK-8 Assay

After transfection, cells were seeded in 96-well plates at a density of 1×10^4^ cells/well. These cells were then maintained in 10% FBS-supplemented DMEM for 24, 48, 72, or 96 h. Cell viability was evaluated by the Cell Counting Kit-8 assay (CCK-8; Dojindo Molecular Technologies, Japan) at each time point. The absorbance was measured at 450 nm by a TECAN infinite M200 plate reader.

### Colony Formation Assay

Forty-eight hours after transfection, cells were seeded in 6-well plates at a density of 1,000 cells/well and cultured at 37°C in a 5% CO_2_ humidified atmosphere. The medium was replaced every other day. The media was removed. Cells were washed twice with PBS after seven days and fixed in methanol for 20 min. Crystal violet (1%) was used to stain the cells for 30 min at room temperature, and then the cells were washed again. Photos were taken with a microscope.

### Flow Cytometric Analysis

Transfected cells were harvested 48 h after transfection. Then, these cells were washed in ice-cold phosphate-buffered saline (PBS), and the apoptosis rate was analyzed with the Alexa Fluor^®^ 488 Annexin V/Dead Cell Apoptosis Kit (Thermo Fisher Scientific, Waltham, MA, USA) according to the manufacturer’s instructions. The cells were analyzed by a Gallios flow cytometer (Beckman Coulter, USA) to quantify the percentage of apoptotic cells. For cell cycle analysis, the transfected cells were fixed in 75% ethanol at 4°C overnight and then stained with reagents from the Cell Cycle Analysis Kit (Beyotime Biotechnology, Jiangsu, China) according to the manufacturer’s instructions. The cells were analyzed on a Gallios flow cytometer (Beckman Coulter, USA) to quantify the proportion of cells in each stage of the cell cycle (S, G1, G2/M).

### Transwell Invasion Assays

Cell invasive capacity was analyzed in Matrigel (BD Biosciences, New Jersey, USA)-coated Transwell chambers (Costar, Corning, NY, USA). Briefly, 200 μl of a cell suspension containing 1 ×10^4^ cells was added to the upper chamber. The lower chamber was covered with DMEM supplemented with 20% FBS. After 24 h incubation, the cells on the top side of the insert membrane were removed by cotton swabs. The cells located on the lower side of the chamber were fixed in 4% paraformaldehyde and stained with 0.1% crystal violet. Finally, the invasive abilities were assessed by counting the invasive cells in images captured under a microscope.

### Wound Healing Assay

Cell migratory capacity was analyzed after transfection. The transfected cells were plated in each well of a 6-well plate and incubated to form 100% confluence. A scratch was made using pipette tips. Next, fresh serum-free medium was replaced. The scratch wound was observed after 24 h, and images were photographed.

### Bioinformatics Prediction

A differential analysis was performed on the gene data of osteosarcoma patients (GSE85537), which was downloaded from the GEO database (https://www.ncbi.nlm.nih.gov/geo/). The target gene of miR-8081 was predicted by the TargetScan website (http://www.targetscan.org/vert_72/). The miRcode website (http://www.mircode.org/) was used to search for potential miRNAs that could be inactivated by NR_136400.

### Western Blot Analysis

Total protein was isolated by means of RIPA buffer (Beyotime Institute of Biotechnology, Shanghai, China). The Bicinchoninic Acid Protein Assay Kit (Pierce, Illinois, USA) was used to quantify the protein concentration. Ten micrograms of protein was subjected to electrophoresis on 8% SDS-PAGE and subsequently transferred onto polyvinylidene difluoride membranes; the membranes were blocked for 2 h with 5% fat-free milk diluted in Tris-buffered saline containing 0.1% Tween 20 (TBST). After incubation with a primary antibody against TUSC5 (1:1,000, Abcam, Cambridge, MA, USA) or against GAPDH (1:2,000, Cell Signaling Technology, Danvers, MA, USA), the membranes were washed thrice with TBST and then incubated with secondary antibody at room temperature for one hour. Protein signals were observed with Pierce™ ECL Western blotting Substrate (Pierce; Thermo Fisher Scientific, Inc.).

### RNA Immunoprecipitation (RIP)

The cells were harvested and resuspended in PBS-based nuclear isolation buffer and then kept on ice for 20 min with frequent mixing. The nuclei were pelleted by centrifugation at 2,500 × g for 15 min. RIPA buffer (1 ml) was used to resuspend the nuclear pellet, which was then equally split into two fractions (for mock and IP). Chromatin was mechanically sheared using a Dounce homogenizer with 15–20 strokes. The nuclear membrane and debris were separated after centrifugation. MS2b binding protein and anti-GFP antibody (10 µg) were added to the supernatant (10 mg) and incubated for 2 h at 4°C. Protein A/G beads (40 µl) were added to the mixture and incubated for 1 h at 4°C. Next, these beads were pelleted and washed in RIPA three times, followed by one wash in PBS. Coprecipitated RNAs were isolated by resuspending the beads in TRIzol reagent.

### Luciferase Reporter Assay

The sequences containing either the wild-type (wt) binding sequence or the mutant (mut) binding sequence for miR-8081 were synthesized by Sangon (Shanghai, China) and inserted into a pGL3 vector (Promega Corporation, Madison, USA). MiR-8081 mimics and the respective reporter plasmids were transfected into U2OS cells using Lipofectamine 2000 according to the manufacturer’s instructions. The Renilla and firefly Luciferase activity was determined 24 h after transfection using a Dual-Luciferase Reporter Assay System (Promega, Madison, WI, USA). Renilla luciferase activity was normalized to that of firefly luciferase.

### Tumor Xenograft Experiment

A total of 20 six-week-old female BALB/c nude mice were purchased from Better Biotechnology Co., Ltd. (Nanjing, China). NR_136400-overexpressing or NR_136400-depleted U2OS cells (1 × 10^7^) were resuspended in 100 μl of phosphate-buffered saline and inoculated subcutaneously into the flanks of nude mice (n=5 for each group). The tumor size was recorded every week, and tumor volume was calculated using the formula: tumor volume = 1/2 ×length ×width^2^. After 3 weeks, all the mice were sacrificed, and the tumor xenografts were excised and weighed. All animal experiments were performed in accordance with guidelines approved by the Animal Care and Use Committee.

### Statistical Analysis

Data are expressed as the mean ± standard deviation (SD) of three independent experiments. Student’s t test was performed to compare the two groups; P < 0.05 was considered statistically significant.

## Result

### LncRNA NR_136400 Is Downregulated in Osteosarcoma

**Figure 1:**
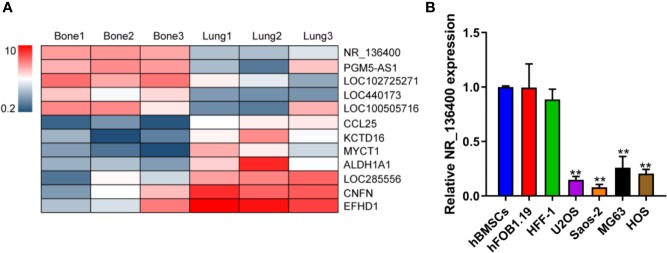
LncRNA NR_136400 is downregulated in osteosarcoma. **(A)** Heatmap of the lncRNA array data for primary osteosarcoma and corresponding lung metastasis. **(B)** The expression of NR_136400 in OS cell lines (U2OS, Saos-2, MG63, and HOS) compared to that in hBMSCs and hFOB1.19 and HFF-1 cells. Data are expressed as the mean ± SD of three independent experiments. “**” indicates P < 0.01.

LncRNA array data for primary osteosarcoma and corresponding lung metastasis from the GEO database are presented in a heatmap, showing that NR_136400 was downregulated in the lung metastatic tumors compared to the primary tumors of osteosarcoma (**Figure 1A**). In the cell lines, the expression of NR_136400 was lower in OS cells (U2OS, Saos-2, MG63, and HOS) than in hBMSCs, hFOB1.19, and HFF-1 cells (**Figure 1B**). Thus, the results indicate that NR_136400 is downregulated in osteosarcoma.

### LncRNA NR_136400 Regulates Cell Viability, Proliferation, and Invasion in Osteosarcoma

**Figure 2:**
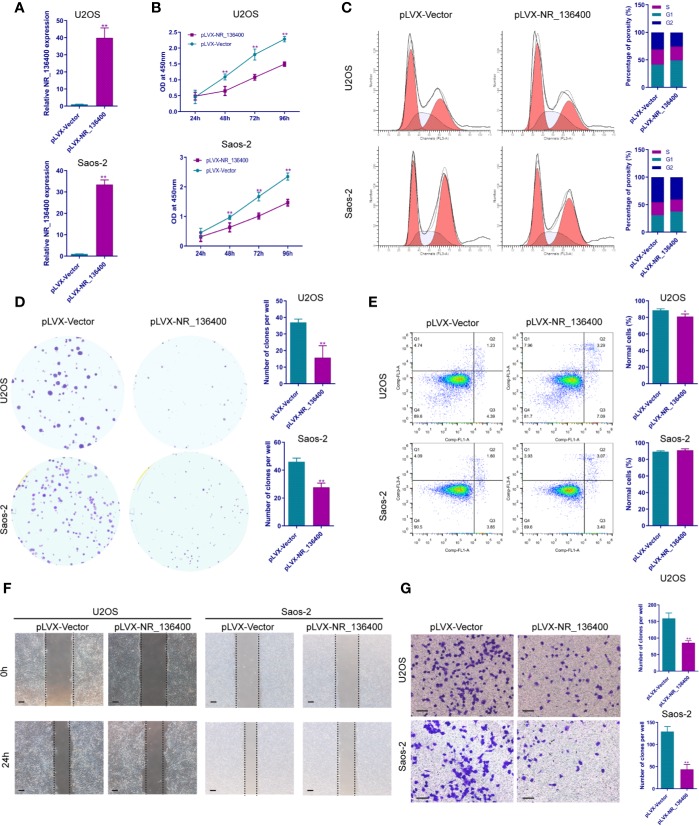
Overexpression of lncRNA NR_136400 inhibits cell viability, proliferation, and invasion in osteosarcoma. **(A)** NR_136400 was efficiently overexpressed in U2OS and Saos-2 cells. **(B)** CCK-8 assay of NR_136400-overexpressing and control U2OS and Saos-2 cells. **(C)** Flow cytometry analysis of the cell cycle for NR_136400-overexpressing and control cells. **(D)** Colony formation assay of NR_136400-overexpressing and control cells in 6-well plates. Representative images (left) and number of colonies (right) are shown. **(E)** Flow cytometry analysis of apoptotic rate for NR_136400-overexpressing and control cells. **(F)** Wound healing assay of NR_136400-overexpressing and control U2OS and Saos-2 cells. Representative images at 0 and 24 h are shown. **(G)** Transwell Matrigel assay of NR_136400-overexpressing and control U2OS and Saos-2 cells. The quantifications of cell invasion are presented as invading cell numbers. Scale bar, 100 μm. All data are expressed as the mean ± SD of three independent experiments. “*” indicates P < 0.05, “**” indicates P < 0.01.

**Figure 3:**
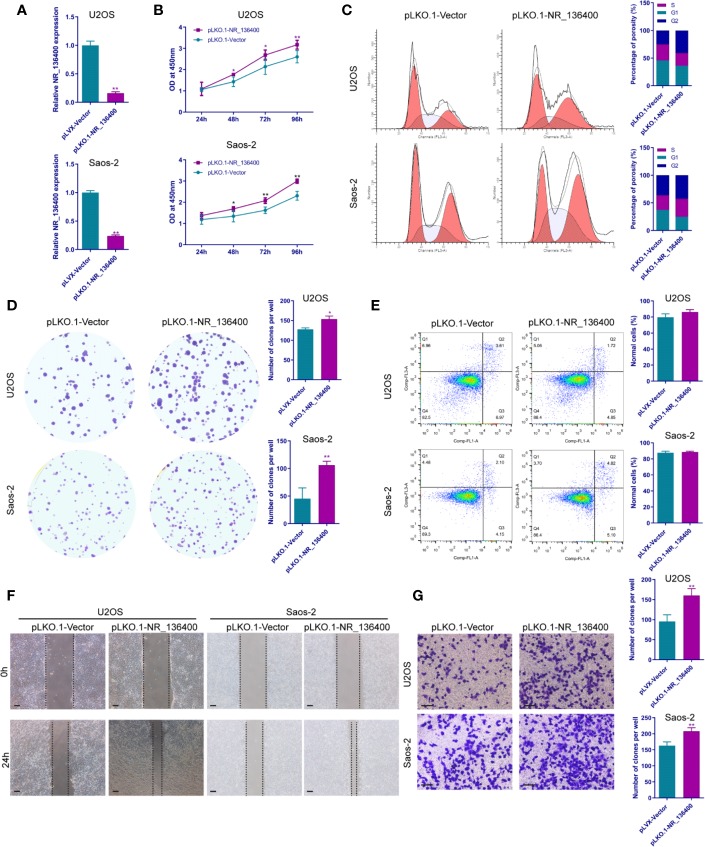
Knockdown of lncRNA NR_136400 facilitates cell viability, proliferation, and invasion in osteosarcoma. **(A)** NR_136400 was efficiently knocked down in U2OS and Saos-2 cells. **(B)** CCK-8 assay of NR_136400-knockdown and control U2OS and Saos-2 cells. **(C)** Flow cytometry analysis of the cell cycle for NR_136400-knockdown and control cells. **(D)** Colony formation assay of NR_136400-knockdown and control cells in 6-well dishes. Representative images (left) and number of colonies (right) are shown. **(E)** Flow cytometry analysis of apoptotic rate for NR_136400-knockdown and control cells. **(F)** Wound healing assay of NR_136400-knockdown and control U2OS and Saos-2 cells. Representative images at 0 and 24 h are shown. **(G)** Transwell Matrigel assay of NR_136400-knockdown and control U2OS and Saos-2 cells. The quantifications of cell invasion are presented as invading cell numbers. Scale bar, 100 μm. All data are expressed as the mean ± SD of three independent experiments. “*” indicates P < 0.05, “**” indicates P < 0.01.

Several *in vitro* experiments were performed to study the biological function of NR_136400 in osteosarcoma. qRT-PCR showed that NR_136400 was efficiently overexpressed (**Figure 2A**) or knocked down (**Figure 3A**) in U2OS and Saos-2 cells. The CCK-8 assay indicated that the viability of NR_136400-overexpressing U2OS and Saos-2 cells was suppressed compared to that in control cells at three time points (48, 72, & 96 h) (**Figure 2B**), while viability was enhanced in NR_136400 knockdown cells (**Figure 3B**). Next, flow cytometry was performed to evaluate how the cell cycle was influenced by dysregulated NR_136400. There was a higher percentage of cells in G1 phase when NR_136400 was overexpressed (**Figure 2C**); conversely, there was a lower percentage of cells in G1 phase when NR_136400 was knocked down (**Figure 3C**). The colony formation assay was performed to evaluate the impact of NR_136400 on tumor cell proliferation and showed that fewer clones formed from NR_136400-overexpressing cells than control cells (**Figure 2D**) and that more clones formed from NR_136400 knockdown cells (**Figure 3D**). According to the flow cytometric apoptosis analysis, U2OS cells had a higher apoptotic rate in the pLVX-NR_136400 group than in the control group, but signals from apoptotic cells and viable cells were similar in both the pLVX-NR_136400 and pLVX-vector groups in Saos-2 cells (**Figure 2E**). When NR_136400 was knocked down, the apoptotic rate was similar between the pLKO.1-NR_136400 and pLKO.1-vector groups in both U2OS and Saos-2 cells (**Figure 3E**). In addition, wound healing and Transwell Matrigel assays were performed to determine the migratory and invasive capacity of the transfected cells. The distance of the scratch wound was significantly larger in NR_136400-overexpressing cells than in control cells (**Figure 2F**), while it was smaller in NR_136400-depleted cells (**Figure 3F**). In the Transwell invasion assay, the number of cells that invaded the chamber in the pLVX- NR_136400 group was significantly less than that in the pLVX- vector group (**Figure 2G**), while it was more in the pLKO.1-NR_136400 group than in the control group (**Figure 3G**). These results indicate that NR_136400 dysregulation modulates cell viability, cell cycle, proliferation, and invasion in osteosarcoma.

### LncRNA NR_136400 Regulates the Migration and Invasion of Endothelial Cells

**Figure 4:**
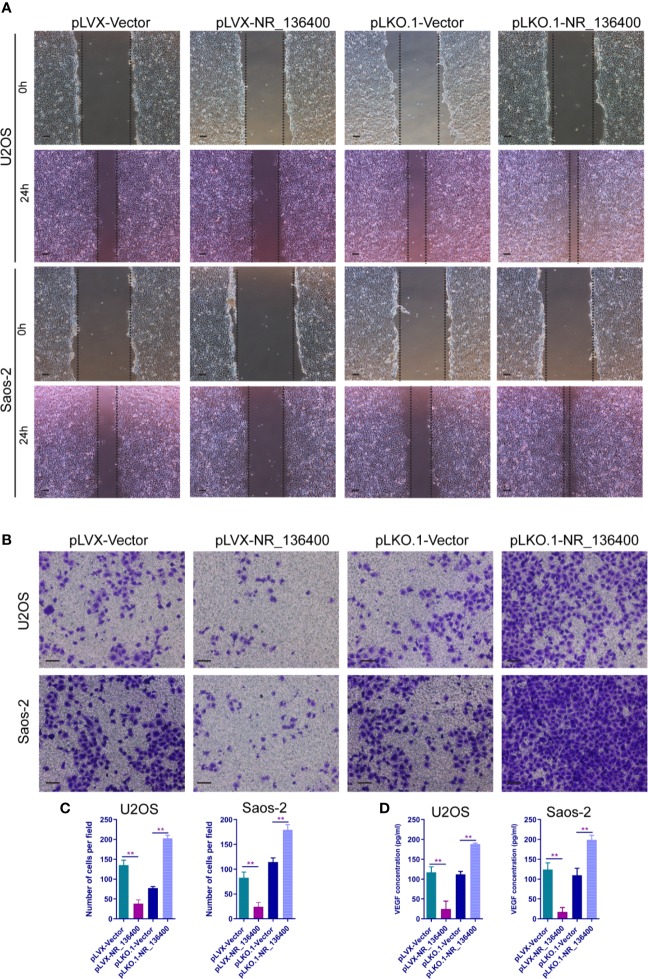
LncRNA NR_136400 regulates the migration and invasion of endothelial cells. **(A)** Wound healing assay of HUVECs cultured with supernatant from NR_136400-overexpressing or NR_136400-depleted U2OS or Saos-2 cells. Representative images at 0 and 24 h are shown. **(B)** Transwell Matrigel assay of HUVECs cultured with supernatant from NR_136400-overexpressing or NR_136400-depleted U2OS or Saos-2 cells. **(C)** The quantifications of invasion are presented as invading cell numbers. **(D)** VEGF concentration as determined by ELISA. Scale bar, 100 μm. All data are expressed as the mean ± SD of three independent experiments. “**” indicates P < 0.01.

To evaluate the impact of NR_136400 on endothelial cells, the culture supernatant of OS cells with NR_136400 overexpression or depletion was collected and added to the media of HUVECs. Wound healing assays showed that there was a larger scratch wound in the pLVX-NR_136400 group and a smaller scratch wound in the pLKO.1-NR_136400 group (**Figure 4A**). In the Transwell Matrigel assay, the number of invading cells was significantly reduced in the pLVX-NR_136400 group compared to the pLVX-vector group, while it was increased in the pLKO.1-NR_136400 group compared to the control group (**Figures 4B, C**). VEGF concentration decreased when HUVECs were cultured in the supernatant of OS cells overexpressing NR_136400 but increased in those cultured in the supernatant of OS cells with depleted NR_136400 (**Figure 4D**). The results indicate that NR_136400 regulates the migration and invasion of endothelial cells.

### Knockdown of lncRNA NR_136400 Facilitates Epithelial-Mesenchymal Transition (EMT)

**Figure 5:**
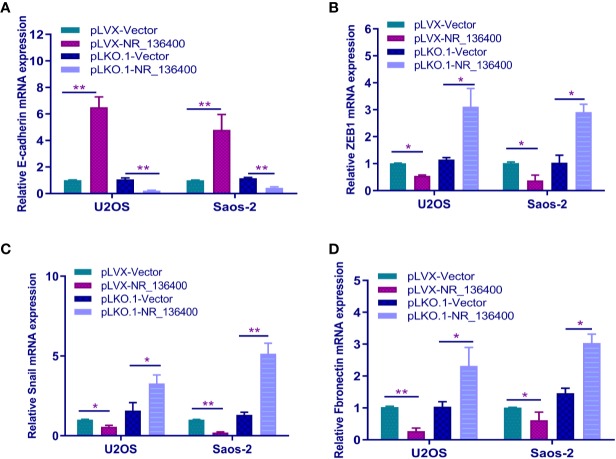
Knockdown of lncRNA NR_136400 facilitates EMT. qRT-PCR analysis of the expression of E-cadherin **(A)**, ZEB1 **(B)**, Snail **(C)**, and fibronectin **(D)** in NR_136400-overexpressing or NR_136400-depleted OS cells (U2OS & Saos-2). All data are expressed as the mean ± SD of three independent experiments. “*” indicates P < 0.05, “**” indicates P < 0.01.

The expression of E-cadherin, ZEB1, Snail, and fibronectin was detected by qRT-PCR to determine the relationship between NR_136400 and EMT. E-cadherin was upregulated when sNR_136400 was overexpressed and downregulated when NR_136400 was knocked down (**Figure 5A**). Meanwhile, the expression of ZEB1 (**Figure 5B**), Snail (**Figure 5C**), and fibronectin (**Figure 5D**) were all downregulated when NR_136400 was overexpressed and upregulated when NR_136400 was knocked down. The results indicate that knockdown of NR_136400 facilitates EMT.

### LncRNA NR_136400 Acts as a Competitive Endogenous RNA of miR-8081

**Figure 6:**
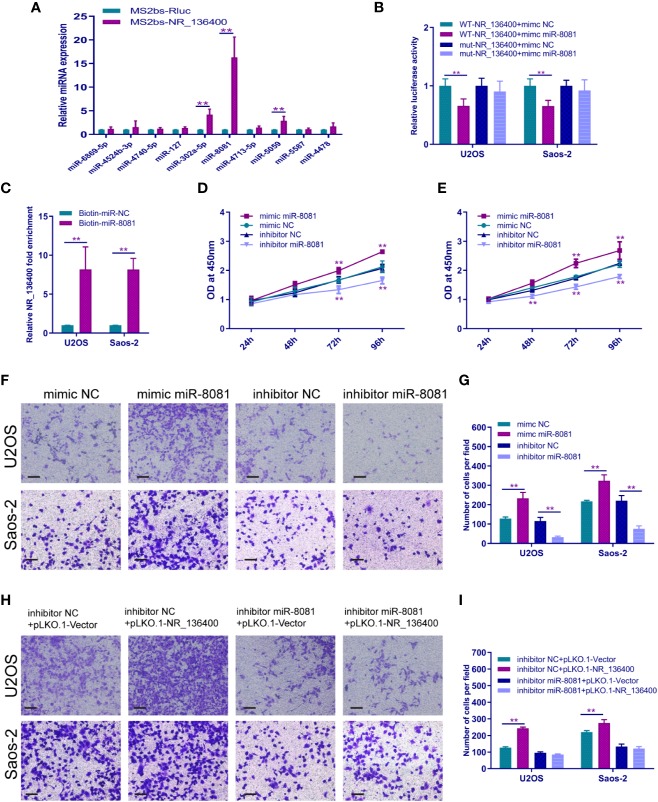
LncRNA NR_136400 acts as a competitive endogenous RNA of miR-8081. **(A)** MS2-based RIP assay with anti-GFP antibody (cross-reactive with YFP) in U2OS cells 48 h after transfection with MS2bp-YFP plasmid along with MS2bs-NR_136400 or MS2bs-Rluc (control vectors). **(B)** Luciferase assay. **(C)** Biotin-miRNA RIP assay. **(D**, **E)** CCK-8 analysis determined the viability of U2OS and Saos-2 cells transfected with miR-8081 mimic or miR-8081 inhibitor. **(F**, **G)** Transwell Matrigel assay was used to determine the invasion ability of U2OS and Saos-2 cells transfected with miR-8081 mimic or miR-8081 inhibitor. **(H**, **I)** Transwell Matrigel assay was used to determine the invasion ability of U2OS and Saos-2 cells cotransfected with miR-8081 inhibitor and pLVX-NR_136400 or miR-8081 inhibitor and pLKO.1-NR_136400. The quantifications of cell invasion are presented as the numbers of invading cells. Data are expressed as the mean ± SD of three independent experiments. “**” indicates P < 0.01.

A panel of miRNAs that could bind to NR_136400 was predicted through miRcode. The MS2b-based RIP assay demonstrated that miR-8081 can bind to NR_136400 (**Figure 6A**). Likewise, the luciferase assay showed that miR-8081 can bind to NR_136400 (**Figure 6B**). In addition, the biotin-miRNA RIP assay showed that miR-8081 can combine with NR_136400 (**Figure 6C**). In the CCK-8 assay, overexpression of miR-8081 increased the viability of U2OS and Saos-2 cells, and suppression of miR-8081 decreased the viability of U2OS and Saos-2 cells (**Figures 6D, E**). In the Transwell assay, overexpression of miR-8081 promoted the invasion of U2OS and Saos-2 cells, and suppression of miR-8081 inhibited the invasion of U2OS and Saos-2 cells (**Figures 6F, G**). Next, we disturbed the function of miR-8081 in U2OS and Saos-2 cells, and the invasion ability promoted by NR_136400 downregulation was reduced. The results indicate that the impact of NR_136400 on the invasion of OS cells is miR-8081 dependent (**Figures 6H, I**).

### LncRNA NR_136400 Regulates the Expression of TUSC5 Through miR-8081

**Figure 7:**
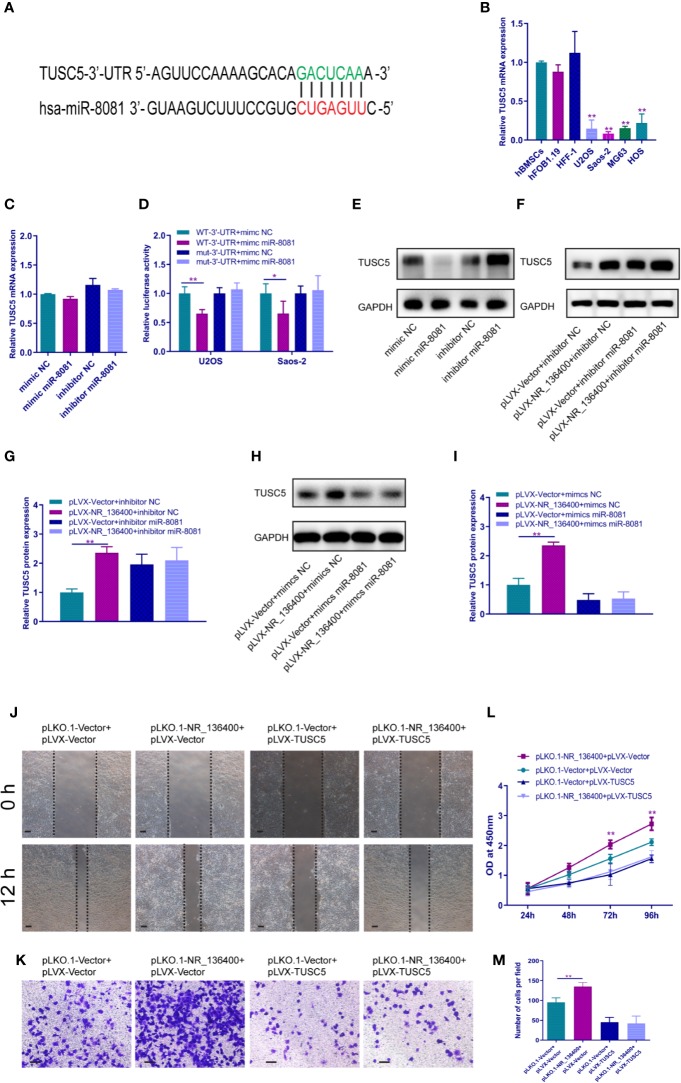
LncRNA NR_136400 regulates the expression of TUSC5 through miR-8081. **(A)** TargetScan showed that TUSC5 was the target of miR-8081. **(B)** qRT-PCR analysis of the expression of TUSC5 in osteosarcoma cell lines (U2OS, Saos-2, MG63, and HOS) compared to that in hBMSCs and hFOB1.19 and HFF-1 cells. **(C)** qRT-PCR analysis of TUSC5 expression in U2OS cells transfected with miR-8081 mimic or miR-8081 inhibitor. The values were normalized to GAPDH mRNA expression. **(D)** Luciferase assay. **(E)** Western blot analysis of the protein expression level of TUSC5 in U2OS cells transfected with miR-8081 mimic or miR-8081 inhibitor. **(F)** Western blot analysis of the protein expression level of TUSC5 in U2OS cells cotransfected with pLVX-NR_136400 and miR-8081 inhibitor. **(G)** Quantitative analysis of the protein expression level of TUSC5 in U2OS cells cotransfected with pLVX-NR_136400 and miR-8081 inhibitor. **(H)** Western blot analysis of the protein expression level of TUSC5 in U2OS cells cotransfected with pLVX-NR_136400 and miR-8081 mimics. **(I)** Quantitative analysis of the protein expression level of TUSC5 in U2OS cells cotransfected with pLVX-NR_136400 and miR-8081 mimics. **(J)** Wound healing assay of NR_136400 knockdown with TUSC5 overexpression in Saos-2 cells. Representative images at 0 and 24 h are shown. **(K)** Transwell Matrigel assay of NR_136400 knockdown with TUSC5 overexpression in Saos-2 cells. **(L)** CCK-8 assay of NR_136400 knockdown with TUSC5 overexpression in Saos-2 cells. **(M)** Quantitative analysis of the Transwell Matrigel assay of NR_136400 knockdown with TUSC5 overexpression in Saos-2 cells. Scale bar, 100 μm. Data are expressed as the mean ± SD of three independent experiments. “*” indicates P < 0.05, “**” indicates P < 0.01.

The binding site of miR-8081 at the 3′-UTR of TUSC5 mRNA was predicted using TargetScan software (**Figure 7A**). The mRNA expression of TUSC5 was detected; it was lower in OS cells than in hBMSCs, hFOB1.19, and HFF-1 (**Figure 7B**). When miR-8081 was overexpressed or knocked down in U2OS cells, the mRNA expression of TUSC5 was not affected (**Figure 7C**). The dual luciferase reporter assay showed that miR-8081 bound to the 3′-UTR of TUSC5 mRNA in U2OS and Saos-2 cells (**Figure 7D**). Western blot analysis showed that TUSC5 protein expression was inhibited when miR-8081 was overexpressed and enhanced when miR-8081 was inhibited (**Figure 7E**). Meanwhile, we found that NR_136400 promoted the expression of TUSC5 protein (**Figure 7F**). Then, we disturbed the function of miR-8081 in U2OS cells; the NR_136400-mediated enhancement of TUSC5 expression was reduced (**Figures 7F–I**), which indicated that the regulation of TUSC5 protein by NR_136400 was miR-8081 dependent. Then, we overexpressed TUSC5 in Saos-2 cells, and NR_136400 knockdown-induced proliferation, migration, and invasion was attenuated (**Figures 7J–M**).

### LncRNA NR_136400 Modulates Tumour Growth in Osteosarcoma

**Figure 8:**
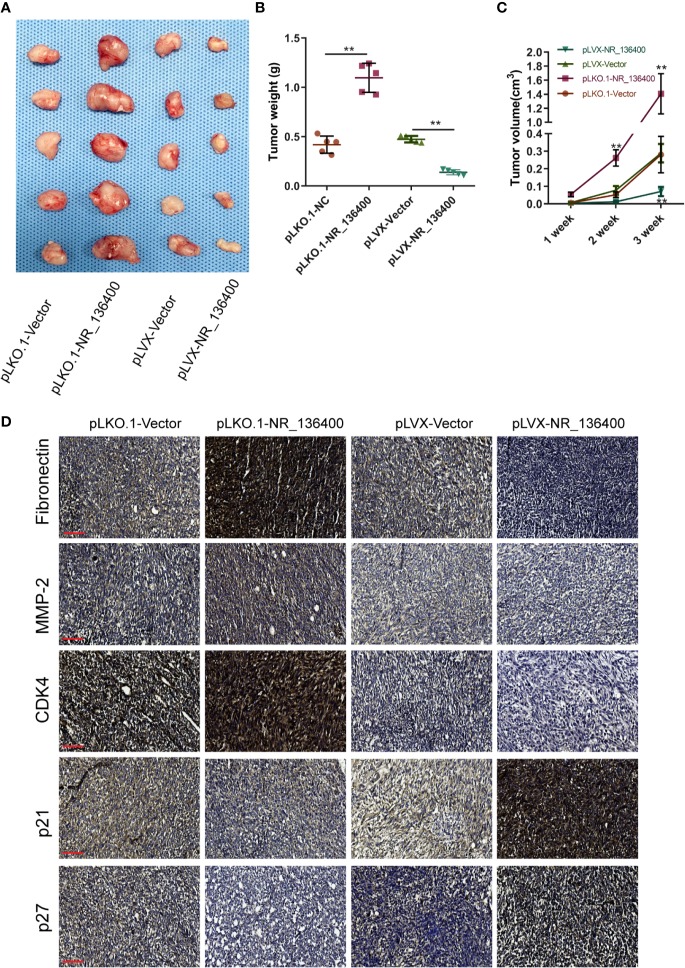
LncRNA NR_136400 modulates osteosarcoma tumor growth. **(A)** The tumor xenografts are shown (n=5 in each group). **(B)** Tumor weight was measured and compared at the end of the 3rd week. **(C)** Tumor volumes were recorded and compared every week. **(D)** Immunohistochemistry was used to detect the expression of Fibronectin, MMP-2, CDK4, p21, and p27. Scale bar, 100 μm. Data are expressed as the mean ± SD. “**” indicates P < 0.01.

*An in vivo* experiment was performed to assess tumor growth. Each group included five nude mice, and the subcutaneous tumor xenografts were compared (**Figure 8A**). The volume of the tumors was recorded every week, and the weight was recorded at the end of the 3rd week. The tumor weight was significantly lower in the pLVX-NR_136400 group than in the pLVX-vector group, while it was significantly higher in the pLKO.1- NR_136400 group than the pLKO.1-vector group (**Figure 8B**). Compared to that in the control group, the tumor volume in the pLVX-NR_136400 group was smaller in the 3rd week, and tumor volume in the pLKO.1-NR_136400 group was larger in the 2nd and 3rd weeks (**Figure 8C**). Immunohistochemistry showed that NR_136400 could promote the expression of p21 and p27 and inhibit the expression of fibronectin, MMP-2 and CDK4 (**Figure 8D**). However, TUSC5 knockdown reduced the suppressive effect of NR_136400 on osteosarcoma (**Figures S1A–C**). The results indicate that overexpression of NR_136400 inhibits osteosarcoma tumor growth.

## Discussion

This study, for the first time, demonstrated the tumor suppressive role of lncRNA NR_136400 and its mechanism in osteosarcoma progression. NR_136400 expression is significantly downregulated in osteosarcoma. In vitro and *in vivo* studies demonstrated that dysregulated NR_136400 regulates the proliferation, invasion, and abnormal apoptosis of OS cells. Moreover, the mechanism of NR_136400 in suppressing osteosarcoma progression has been validated. NR_136400 regulates TUSC5 expression by competitively binding to miR-8081.

LncRNAs are long non-coding regulatory RNAs that are longer than 200 nucleotides (Lee, 2012). The role of lncRNAs in the regulation of cellular processes and cancerous progression is gaining attention (Chi et al., 2019; Lv and Huang, 2019). Zhu et al. (2019) reported that the long noncoding RNA CTC-297N7.9 was downregulated in hepatocellular carcinoma and predicted poor prognosis in these patients. LncRNA PCAT-1 was reported as a tumor suppressor in ovarian cancer, and silencing PCAT-1 caused suppression of proliferation, migration, and invasion and promotion of apoptosis (Min and Chu, 2019). In osteosarcoma, lncRNA SOX2-OT is recognized as a novel prognostic biomarker for osteosarcoma patients and regulates osteosarcoma cell proliferation and motility by modulating SOX2 (Wang et al., 2017). In our study, NR_136400 was first investigated in cancer. We found that NR_136400 was downregulated in primary osteosarcoma samples and was expressed at even lower levels in corresponding lung metastasis samples. The biological role of NR_136400 has been validated, indicating that NR_136400 is a tumor suppressor in osteosarcoma. We found that NR_136400 had a significant effect on the proliferation, migration, and invasion of osteosarcoma cells but had little effect on the apoptosis of osteosarcoma cells. Overexpression of NR_136400 promoted the apoptosis of U2OS cells but had no effect on the apoptosis of Saos-2 cells. It is possible that NR_136400 has little effect on the apoptosis of cells and that the main function is to affect the proliferation, migration, and invasion of tumor cells attack.

LncRNAs have been reported in different types of cancer; however, the mechanism by which lncRNAs mediate the formation and progression of cancerous diseases has not been clearly elucidated. In the present study, lncRNA NR_136400 was confirmed to competitively bind with miR-8081, indicating that NR_136400 acts as a ceRNA of miR-8081 to regulate the protein expression of TUSC5 and then regulate the biological function of osteosarcoma. Thus, NR_136400 functions at the transcriptional level to regulate the progression of osteosarcoma.

The relevance of NR_136400 to the EMT process was also explored in our study. EMT plays a critical role in tumor growth, which facilitates the transformation of epithelial cells into mesenchymal cells to reduce intercellular attachments, reduce cell polarity, and improve motility and migration (Cheng et al., 2019; Williams et al., 2019). Previous studies validated that cancer metastasis is associated with EMT (Angelucci et al., 2012; Labernadie et al., 2017). E-cadherin is known as one of the hallmarks of EMT (De Craene and Berx, 2013). It has been recently demonstrated that a reduction in E-cadherin expression is related to tumor growth, differentiation, growth and metastasis, suggesting a possible role of E-cadherin in cancer progression (Samatov et al., 2013; Choupani et al., 2018). Our results showed that E-cadherin expression was significantly increased when NR_136400 was overexpressed in U2OS and Saos-2 cells. The results suggest that the silencing of NR_136400 in OS cells promotes the EMT process, indicating that silenced NR_136400 facilitates the metastatic process of OS.

## Conclusion

Our study identified a novel lncRNA, NR_136400, that regulates the proliferation and invasion of osteosarcoma. NR_136400 acts as a competitive endogenous RNA of miR-8081 and then mediates TUSC5 expression. Our study has expanded our understanding of the role of lncRNAs in osteosarcoma and provides a strategy for lncRNA-directed therapeutics against osteosarcoma.

## Data Availability Statement

The raw data supporting the conclusions of this article will be made available by the authors, without undue reservation, to any qualified researcher.

## Ethics Statement

The animal study was reviewed and approved by Animal Care and Use Committee of Second Military Medical University.

## Author Contributions

YG and QG designed the experiments. LL and MZ performed the experiments and acquired the data. YG and QG analyzed the data. YG and QG supervised the project. YG, QG, and XW wrote the manuscript.

## Conflict of Interest

The authors declare that the research was conducted in the absence of any commercial or financial relationships that could be construed as a potential conflict of interest.

## Supplementary Material

Figure S1 LncRNA NR_136400-mediated modulation of osteosarcoma tumor growth is dependent on TUSC5. **(A)** The tumor xenografts are shown (n=5 in each group). **(B)** Tumor weight was measured and compared at the end of the 3rd week. **(C)** Tumor volumes were recorded and compared every week. Data are expressed as the mean ± SD. “**” indicates P < 0.01.
