# Supplementary figures and images for: LncRNA NR_136400 Suppresses Cell Proliferation and Invasion by Acting as a ceRNA of TUSC5 That Is Modulated by miR-8081 in Osteosarcoma
Source: Front Pharmacol. 2020 May 15;11:641. doi: 10.3389/fphar.2020.00641 (PMC7242660; doi:10.3389/fphar.2020.00641)

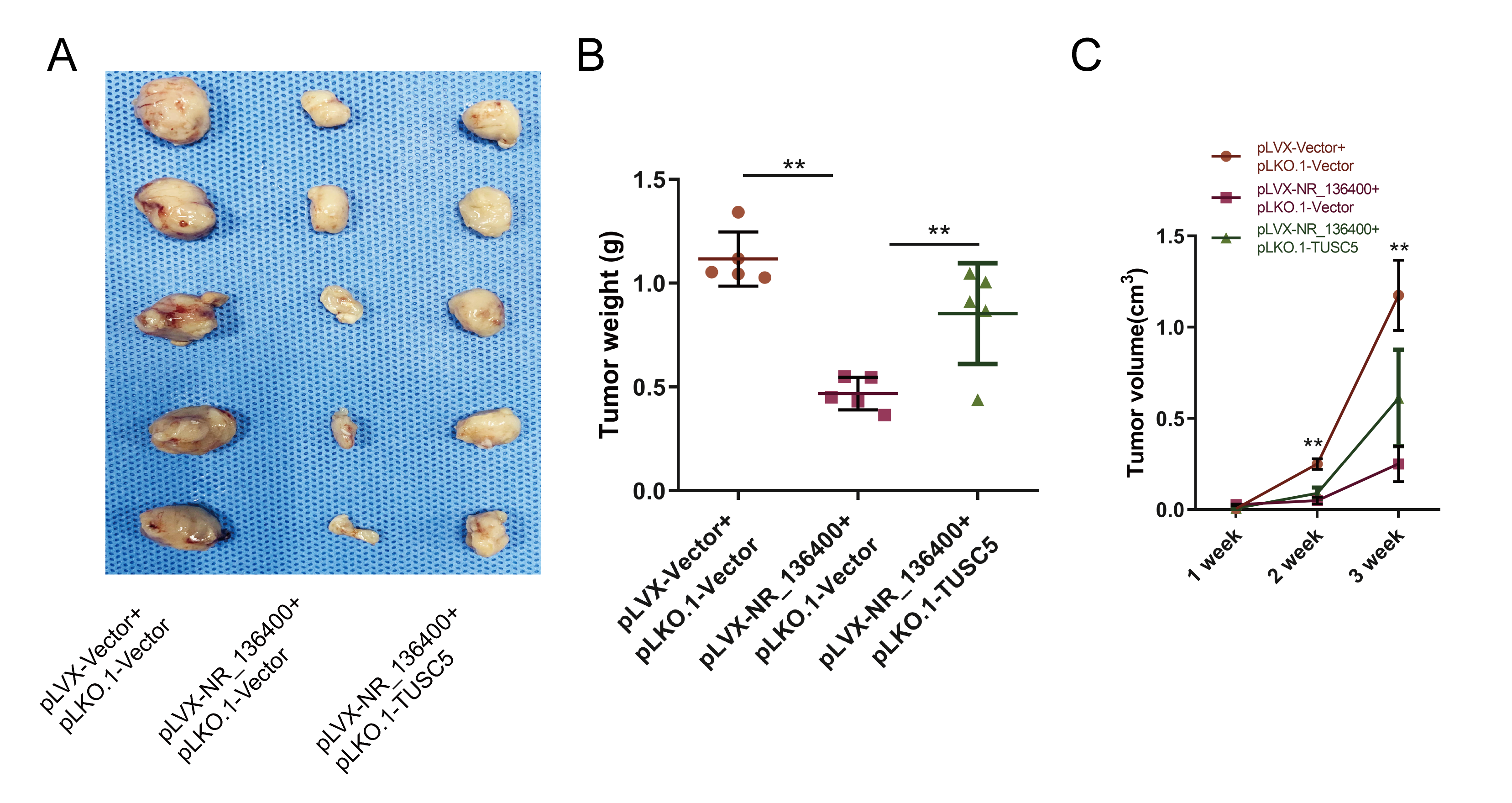

Supplement: Figure S1 — LncRNA NR_136400-mediated modulation of osteosarcoma tumor growth is dependent on TUSC5. (A) The tumor xenografts are shown (n=5 in each group). (B) Tumor weight was measured and compared at the end of the 3rd week. (C) Tumor volumes were recorded and compared every week. Data are expressed as the mean ± SD. “**” indicates P < 0.01. [file Image_1.tif]
